# Refining Open-World Game and Nostalgic Film Interventions for Broader and More Reliable Therapeutic Impact

**DOI:** 10.2196/82157

**Published:** 2025-10-01

**Authors:** Chenlei Sun, Ke Meng

**Affiliations:** 1 Department of Music, Yeungnam University, 280 Daehak-Ro, Gyeongsan, 38541, Republic of Korea, 82 01084423017; 2 Department of Global Convergence, Kangwon National University, Chuncheon, Republic of Korea

**Keywords:** open-world games, nostalgia, lab experimental study, postgraduate students, My Neighbor Totoro, Kiki's Delivery Service, Nintendo

We read with great interest the recent study by Arigayota et al [1] that explored the interplay between immersive gameplay, nostalgic film experiences, and psychological well-being. As art therapists, we find this approach inspiring, as it bridges creative media and emotional health. However, in considering how these findings might translate to real-world interventions, several methodological aspects deserve discussion.

First, the recruitment strategy—inviting graduate students to volunteer—may inadvertently favor individuals already inclined toward gaming, Studio Ghibli films, or similar forms of media engagement. Such self-selection could produce a sample with higher baseline openness to immersive and nostalgic experiences, potentially amplifying the observed effects. In therapeutic practice, participants often have varied or even limited familiarity with certain media. Future research could broaden recruitment to include individuals with differing media backgrounds and use screening tools to capture baseline engagement preferences. This would help clarify whether the observed benefits generalize beyond those already predisposed to enjoy these stimuli.

Second, the intervention’s brevity—30 minutes of gameplay and approximately 7 minutes of film viewing—captures only a snapshot of the possible psychological impact. While short sessions are pragmatic in experimental settings, art therapy often relies on sustained engagement to foster deeper emotional processing and behavioral change [2,3]. Extending the intervention into a multi-session format, spaced over days or weeks, could reveal whether benefits in calmness, exploration, and meaning consolidate into more enduring well-being gains. Moreover, varying session length could help identify the minimal “dose” necessary to elicit therapeutic outcomes, offering valuable guidance for applied settings.

Third, while the study accounted for familiarity and liking of the media, other potential confounding variables—such as the participants’ mood before the session, recent stressors, sleep quality, or social interactions—were not fully controlled. These factors can significantly influence immediate emotional responses and may have interacted with the intervention’s effects. In a therapeutic context, we routinely monitor and adapt to such variables, as they shape how individuals respond to creative activities. Incorporating pre-session mood assessments or controlling for recent life events could help disentangle the intervention’s unique contributions from the influence of transient emotional states.

In summary, this study offers a valuable foundation for integrating immersive and nostalgic media into well-being interventions. Addressing self-selection bias, exploring longer or repeated exposures, and more comprehensively controlling for confounding factors could strengthen future research and enhance its applicability in therapeutic settings. By refining these aspects, the promising combination of open-world gaming and nostalgic film could evolve into a versatile, evidence-based tool for supporting emotional health across diverse populations.
